# Attenuation of ischemia–reperfusion injury by intracoronary chelating agent administration

**DOI:** 10.1038/s41598-022-05479-2

**Published:** 2022-02-08

**Authors:** Donghoon Han, Si-Hyuck Kang, Chang-Hwan Yoon, Tae-Jin Youn, In-Ho Chae

**Affiliations:** 1 Department of Internal Medicine, Cardiovascular Center, Hallym University Kangnam Sacred Heart Hospital, Seoul, Korea; 2 Department of Internal Medicine, Seoul National University College of Medicine, Cardiovascular Center, Seoul National University Bundang Hospital, 82 Gumiro 173, Bundang, Seongnam, Republic of Korea

**Keywords:** Mechanisms of disease, Interventional cardiology

## Abstract

Ischemia–reperfusion (IR) injury accelerates myocardial injury sustained during the myocardial ischemic period and thus abrogates the benefit of reperfusion therapy in patients with acute myocardial infarction. We investigated the efficacy of intracoronary ethylenediaminetetraacetic acid (EDTA) administration as an adjunctive treatment to coronary intervention to reduce IR injury in a swine model. We occluded the left anterior descending artery for 1 h. From the time of reperfusion, we infused 50 mL of EDTA-based chelating agent via the coronary artery in the EDTA group and normal saline in the control group. IR injury was identified by myocardial edema on echocardiography. Tetrazolium chloride assay revealed that the infarct size was significantly lower in the EDTA group than in the control group, and the salvage percentage was higher. Electron microscopy demonstrated that the mitochondrial loss in the cardiomyocytes of the infarcted area was significantly lower in the EDTA group than in the control group. Echocardiography after 4 weeks showed that the remodeling of the left ventricle was significantly less in the EDTA group than in the control group: end-diastolic dimension 38.8 ± 3.3 mm vs. 43.9 ± 3.7 mm (n = 10, *p* = 0.0089). Left ventricular ejection fraction was higher in the EDTA group (45.3 ± 10.3 vs. 34.4 ± 11.8, n = 10, respectively, *p* = 0.031). In a swine model, intracoronary administration of an EDTA chelating agent reduced infarct size, mitochondrial damage, and post-infarct remodeling. This result warrants further clinical study evaluating the efficacy of the EDTA chelating agent in patients with ST-segment elevation myocardial infarction.

## Introduction

In-hospital mortality in patients with ST segment elevation myocardial infarction (STEMI) mortality has decreased because of advanced medical treatments and the advent of reperfusion therapy^1^. However, left ventricular remodeling and heart failure after myocardial infarction remains a common problem with high mortality^2^. Therefore, further treatment to prevent remodeling and heart failure after myocardial infarction is needed, given the lack of improvement in their long-term prognosis. Ischemia–reperfusion (IR) injury is considered to accelerate myocardial injury sustained during the myocardial ischemic period after reperfusion therapy such as primary percutaneous coronary intervention^3^. Three mechanisms inducing reperfusion injury are reactive oxygen species, Ca^2+^ overload, and pH correction after reperfusion, all of which contribute to the opening of the mitochondrial permeability transition pore (mPTP), leading to mitochondrial dysfunction and myocardial necrosis. This is an acute process occurring within several minutes to 30 min after reperfusion^4^. In the real clinical setting, many patients complained of sudden, serious chest pain within a few minutes after reperfusion in primary percutaneous coronary intervention, suggesting the existence of IR injury.

Ischemic conditioning offers a powerful endogenous cardioprotective strategy that prevents mitochondrial dysfunction by reducing the size of MI in patients with STEMI undergoing reperfusion. However, clinical studies using various types of ischemic conditioning have produced mixed results^5^. Although clinical cardioprotective research has been challenging, novel therapies are still needed.

Ethylenediamine tetraacetic acid (EDTA) is a synthesized amino acid with strong, non-specific chelating properties for valences of + 2 to + 6 including calcium, reduces tissue calcium content, and facilitates urinary excretion of calcium^6^. Previous studies reported that mPTP was inhibited by scavenging calcium ions in an in vitro model of IR injury^7,8^.

We investigated whether intracoronary administration of EDTA after reperfusion could reduce calcium in the injured tissue, prevent the transition of mPTP and mitochondrial dysfunction, and thereby reduce IR injury in a swine IR injury model.

## Results

### Effects of EDTA on acute ischemia–reperfusion injury

All pigs were 4–5 months old. The initial body weights were not significantly different between the EDTA and control groups (22.5 ± 5.8 vs. 22.1 ± 4.62.5, p = 0.86).

**Figure 1:**
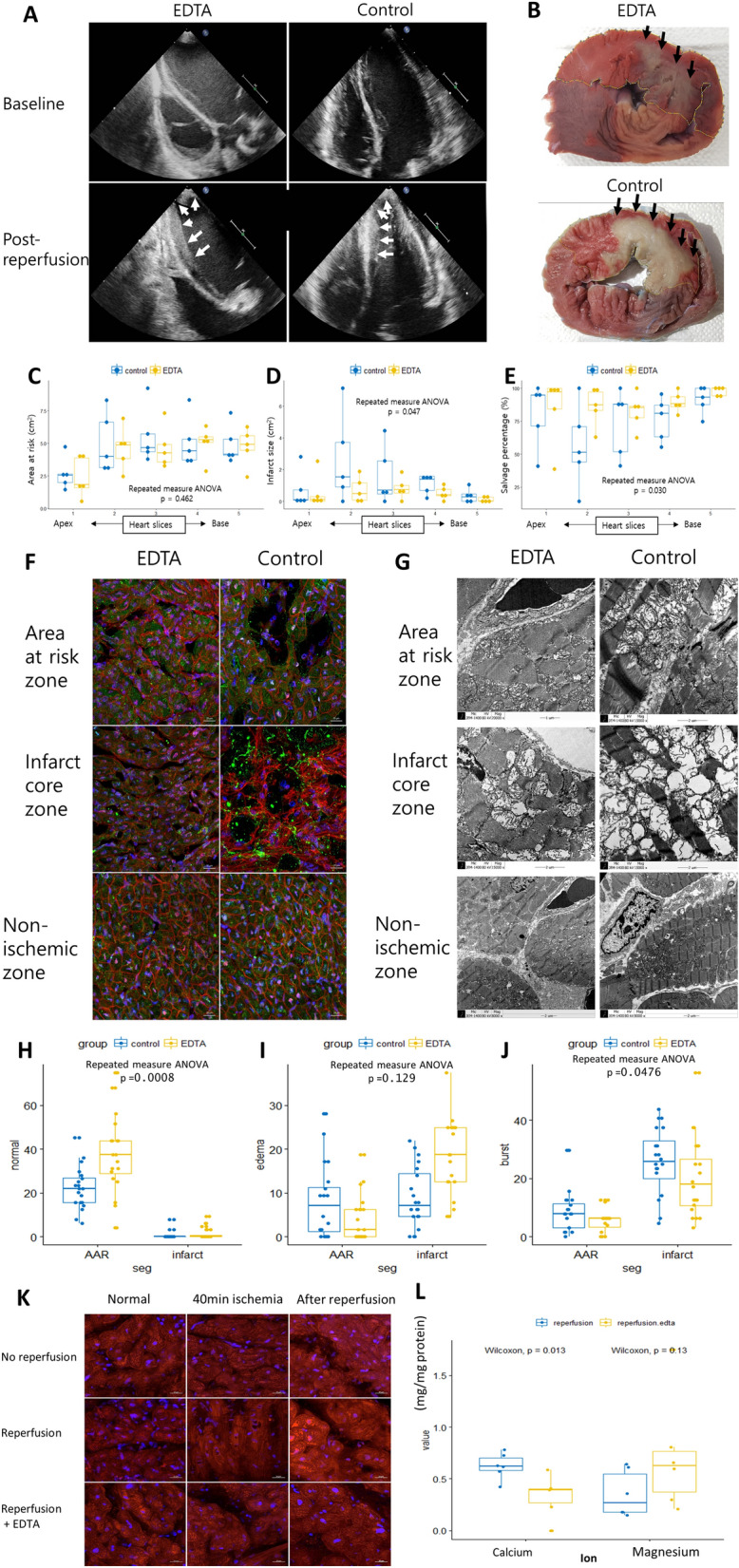
Effect of EDTA on acute reperfusion injury. (**A**) Baseline and post-reperfusion echocardiography in the EDTA and control groups. Arrows indicate myocardial edema after reperfusion in the EDTA group. (**B**) Area at risk (AAR) was delineated in the area without a blue tinge (yellow line). Infarct size (IFS) was measured in the area with a whitish color within the AAR (arrows). (**C–E**) IFS was smaller in the EDTA group than in the control group. Salvage percentage was higher in the EDTA group. (**F**) Myocardial tissues sections were stained with WGA(red), heavy chain cardiac myosin(green), DAPI(blue) and observed by confocal microscopy. Compared to the compact cardiomyocytes in the non-ischemic zone, interstitial space in the area at risk zone was increased due to myocardial edema and destroyed cardiomyocytes in the infarct core zone were observed. (**G**) Myocardial tissues using electron microscopy. Compared to the compact and dense mitochondria in the non-ischemic zone, mitochondria were swollen and edematous in areas at risk or burst and left as empty spaces in the infarct core zone. (**H–J**) Percentage of mitochondria in three different morphologies (normal/edema/burst) in the AAR zone. and infarct core zone. (**K**) Myocardial tissues were stained using MitoSOX to demonstrate the ROS status during reperfusion. The intensity of MitoSOX staining was more preserved in the EDTA group than in the control group. (**L**) Compared to calcium and magnesium concentrations between the two groups. The level of calcium was significantly lower in the EDTA group; otherwise, the level of magnesium was not significantly different.

After occlusion, akinesia in the apex and mid anterior and septal walls on echocardiography and ST segment elevation were noted (Supplementary video). After reperfusion, the echogenicity of the akinetic segments increased within 5 min and myocardial edema gradually increased until 20 min after reperfusion (Fig. 1A and supplementary video). We measured AAR, IFS, and salvage percentage using postmortem 5 heart slices in each pig (Fig. 1B–E). AAR was not different between the groups (*p* = 0.462) (Fig. 1C). However, IFS was significantly lower and, thus, the salvage percentage was significantly higher in the EDTA group than in the control group (Fig. 1D,E).

To obtain a mechanistic insight into the reduction of reperfusion injury, we examined the immunofluorescent staining of the myocardial tissue and transmission electron microscopy of the mitochondria in the non-ischemic, AAR, and infarct core zones after reperfusion. Compared to the compact cardiomyocytes in the non-ischemic zone, interstitial space in the area at risk zone was increased due to myocardial edema and destroyed cardiomyocytes in the infarct core zone (Fig. 1F). Compared to the compact and dense mitochondria in the non-ischemic zone, mitochondria were swollen and edematous in areas at risk or burst and left as empty spaces in the infarct core zone (Fig. 1G). These changes were more prominent in the control group. The percentage of normal mitochondria in the AAR zone was significantly higher (p = 0.0006) and the percentage of burst mitochondria in the infarct core zone was significantly lower (p = 0.0476) in the EDTA group than in the control group (Fig. 1H–J).

### The ROS status during reperfusion

We stained the myocardium biopsy tissues using MitoSOX™ to detect and compare the Reactive oxygen species (ROS) status between the two groups. Confocal microscopy showed that the intensity of MitoSOX™ staining was preserved in the EDTA group. However, MitoSOX™ staining was increased in the control group (Fig. 1K).

### Calcium and magnesium concentration

To demonstrate the mechanism of EDTA, we measured calcium (CA) and magnesium (MG) concentrations from the myocardium. The level of CA was significantly lower in the EDTA group than in the control group (*p* = 0.013, Fig. 1L). Otherwise, the level of MG was higher in the EDTA group than in the control group, but the difference was not statistically significant (*p* = 0.13, Fig. 1L).

### Effects of EDTA on LV remodeling after ischemia–reperfusion injury

**Figure 2:**
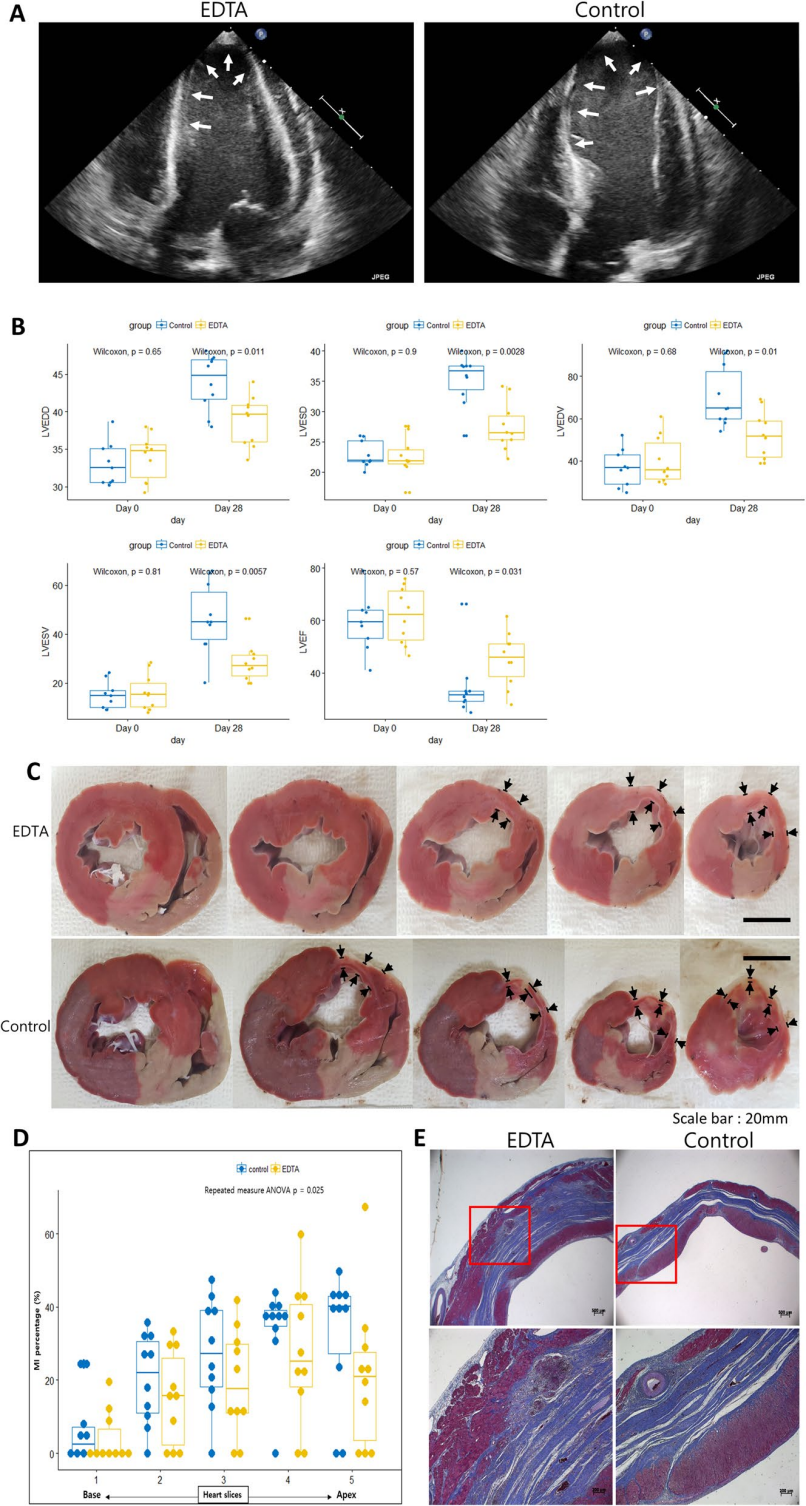
Effect of EDTA on LV remodeling. (**A**) representative echocardiographic view of the infarcted heart on day 28. Arrows, infarcted segments (**B**) The difference between left ventricular end-diastolic diameter (LVEDD), left ventricular end-systolic diameter (LVESD), left ventricular end-diastolic volume (LVEDV), left ventricular end-systolic volume (LVESV), and left ventricular ejection fraction (LVEF) by volumetry at baseline and on day 28. (**C**) Representative heart slices for the pathologic evaluation of post-infarct remodeling. Arrows indicate infarcted segments and thickness of segments. (**D**) Percentage of infarcted area in the slices. (**E**) Representative images of Masson’s trichrome staining of the infarcted segment in each group. Rectangles in the upper panel coincide with the lower panel.

After 28 days, the body weight increased in both groups (29.2 ± 5.4 vs. 26.9 ± 4.5, p = 0.49). Myocardial thinning, infarct expansion, and left ventricular dilation occurred in both groups to a different degree (Fig. 2A). All echocardiographic measurements did not differ between the groups on day 0 (Fig. 2B and Table 1). However, left ventricular dimensions and volumes were significantly lower in the EDTA group than in the control group, and left ventricular ejection fraction was significantly higher in the EDTA group on day 28. did not differ between the groups (Fig. 2B). We also measured the length of the thinned and akinetic LV walls in a subcostal view on day 28. The length of the thinned and akinetic walls was significantly lower in the EDTA group than in the control group (Table 1).

**Table 1:** Echocardiographic parameters of the EDTA and control groups.

		EDTA (n = 10)	Control (n = 10)	*p* value
Day 0	LVEDD (mm)	34 ± 3.1	33.0 ± 2.9	0.6532
	LVESD (mm)	22.6 ± 3.2	22.9 ± 2.2	0.9023
	LVEDV (mL)	40.0 ± 11.2	36.8 ± 9.0	0.683
	LVESV (mL)	16.2 ± 7.3	15.0 ± 5.7	0.8061
	LV EF (%)	61.9 ± 10.7	59.2 ± 10.7	0.5675
Day 28	LVEDD (mm)	38.8 ± 3.3	43.9 ± 3.7	0.0089
	LVESD (mm)	27.5 ± 4.0	35.2 ± 4.1	0.0028
	LVEDV (mL)	52.1 ± 11.4	70.2 ± 14.3	0.0101
	LVESV (mL)	28.3 ± 7.9	46.5 ± 14.3	0.0057
	LV EF (%)	45.3 ± 10.3	34.4 ± 11.8	0.031
	Thinning (mm)	44.2 ± 1.3	86.8 ± 10.5	0.003
	Total length (mm)	139.0 ± 5.4	141.2 ± 14.5	0.76
	Thinning ratio (%)	31.8 ± 0.6	61.5 ± 5.9	0.002

*LVEDD* left ventricular end-diastolic diameter, *LVESD* left ventricular end-systolic diameter, *LVEDV* LV end-diastolic volume, *LVESV* left ventricular end-systolic volume, *LVEF* left ventricular ejection fraction by volumetric calculation.

Moreover, we also measured the infarcted area of the slices of the harvested heart (Fig. 2C). The percentage of infarcted area in the slices was significantly lower in the EDTA group than in the control group (Fig. 2D). The thickness of the infarcted segment was higher and viable myocardium was more abundant in the EDTA group than in the control group (Fig. 2C,E).

## Discussion

In this study, we demonstrated that intracoronary administration of an EDTA-based chelating solution as a postconditioning agent in the setting of catheter-based reperfusion therapy reduces ischemia–reperfusion injury by reducing mitochondrial damage and thus the infarct size. Moreover, the EDTA-based chelating solution decreased LV remodeling and preserved LV systolic function.

Reperfusion injury is mediated by reactive oxygen species, Ca^2+^ overload, sudden pH correction, and opening of the mitochondrial permeability transition pore (mPTP)^3^. Open mPTP leads to ATP loss and cell death^9^. Hence, mPTP plays a key role in lethal reperfusion injury; thus, it is a new target to protect the ischemic myocardium from reperfusion injury. Various strategies to reduce IR injury, including pharmacologic or balloon occlusive postconditioning, showed disappointing results^10–12^.

Disodium ethylenediaminetetraacetic acid (EDTA) is a chelating agent that forms strong covalent bonds with calcium and increases the urinary excretion of calcium. It has been reported to reduce tissue calcium and improve symptoms in patients with severe angina^13,14^. However, it was uncertain whether EDTA chelation was effective for cardiovascular disease. Meanwhile, a trial reported that administration of intravenous EDTA solution as a chelating agent decreased the risk of adverse cardiovascular outcomes among stable patients with previous myocardial infarction^15^. Although an accurate mechanism for the effect of EDTA was not established and the authors only assumed an anti-oxidant effect, the trial confirmed the efficacy and safety of EDTA in patients with cardiovascular disease.

In this study, we investigated whether intracoronary artery administration of an EDTA chelating agent during reperfusion reduces IR injury in a swine ischemia–reperfusion model. Forty minutes of myocardial ischemia followed by reperfusion leads to an 18-fold increase in calcium in the damaged tissue^4^. In a previous study, intracoronary infusion of ethylene glycol tetraacetic acid (EGTA), which is a chelating agent similar to EDTA, reduced free coronary calcium concentrations during a 45-min reperfusion^16^. Therefore, EDTA may have similar efficacy in reducing myocardial calcium content. EGTA significantly decreased infarct size and improved regional systolic shortening until 3 days after ischemia–reperfusion injury. However, whether EGTA could reduce mitochondrial dysfunction and ultimately lead to long-term improvement of LV systolic function and remodeling was not investigated in that study. EDTA rapidly closed mPTP in an in vitro model of ischemia–reperfusion injury by free calcium sequestration^17^. In the present study, we clearly found that intracoronary EDTA protected myocardial mitochondria during reperfusion, which might be a cornerstone of the effects in that of EDTA chelating solution reduced the infarct size and left ventricular remodeling associated with reperfusion injury. Thus, we hypothesized that the EDTA chelating solution could preserve the left ventricular systolic function in a large animal IR injury model.

### Study limitations

This study has a few limitations. First, the number of animals was not large. Some of the significant findings were derived from repeated measures within subjects. However, the most important findings, echocardiographic evaluations, which were a single measurement for each subject, resulted in significant differences with very low alpha error. Therefore, it is not probable that the main findings of this study may come incidentally. Second, the EDTA solution mainly included disodium EDTA; however, it also consisted of various materials, which possibly influenced myocardial protection. Third, we did not measure mitochondrial calcium transients in live swine hearts. Therefore, mitochondrial protection may not be related to calcium handling by EDTA. Live imaging can be performed in mice using either two-photon microscopy or bioluminescence imaging^18^. However, it is technically difficult in a large animal model. Assays based on in vitro preparation of mitochondria or culture cells are available. However, these assays are limited to understanding the actual process in vivo because of mitochondrial membrane potential and spatio-temporal constraints. Lastly, intracoronary EDTA administration had potential hazards to be applied in a clinical setting. EDTA could potentially cause severe hypocalcemia and death when misused^19^. However, EDTA was associated with few side effects, as used in the TACT trial^15^. Although the dose was one-fifth of that in the TACT trial, the administrative route and the clinical setting were different. We occasionally encountered ventricular tachycardia or ventricular fibrillation during EDTA infusion. However, the electrocardioversion and efforts to stabilize the pigs were always successful, and there were no cases of intractable or fatal tachyarrhythmia. Nevertheless, safety should be re-evaluated for further clinical studies.

In conclusion, intracoronary administration of an EDTA-based chelating solution as a postconditioning agent showed a protective effect against myocardial IR injury and left ventricular remodeling in a swine model. Further clinical studies are warranted to prove its efficacy and safety in patients with acute myocardial infarction.

## Material and methods

### EDTA chelating agent

EDTA-based chelating solution consisted of 3 g of disodium EDTA, 7 g of ascorbic acid, 2 g of magnesium chloride, 100 mg of procaine hydrochloride, 2500 U of unfractionated heparin, 2 mEq of potassium chloride, 840 mg of sodium bicarbonate, 250 mg of pantothenic acid, 100 mg of thiamine, 100 mg of pyridoxine, and sterile water to make up 500 mL of solution^15^.

### Animal preparation

The day before the experiment, male crossbred swine (*n* = 30; weight, 17–35 kg) were fed with aspirin (300 mg) and clopidogrel (300 mg). On the day of the experiment, the swine were premedicated with atropine sulfate (0.05 mg/kg, intramuscularly) and subsequently anesthetized with Zoletil (5 mg/kg) and xylazine (4.4 mg/kg, intramuscularly), intubated, and ventilated with room air and isoflurane. We inserted a 6-Fr. sheath via the right femoral artery via ultrasound-guided puncture.

### Ischemia and reperfusion injury with or without the chelation postconditioning procedure

**Figure 3:**
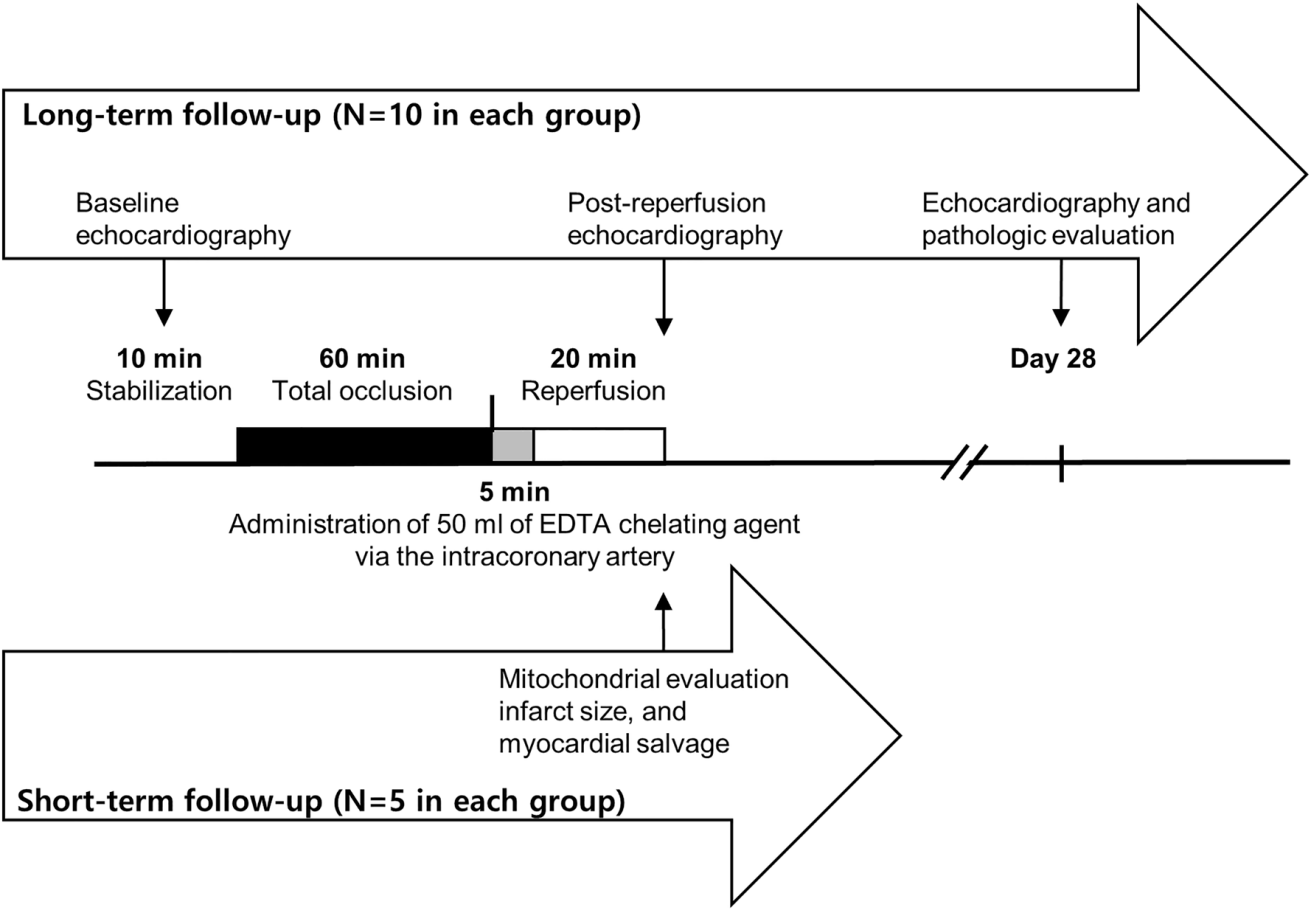
Schematic study flow.

The animals received heparin (5000 U, intravenously) prior to coronary artery catheterization. We cannulated the left coronary artery with a guiding catheter (Judkins right 4) and performed coronary angiography. Thereafter, we inserted a coronary guidewire (0.014″) and 3.0 × 15 mm over-the-wire balloon to the mid-left anterior descending artery. To induce myocardial ischemia, we inflated the balloon and totally occluded the artery for 1 h. Subsequently, we deflated the balloon and secured adequate coronary flow to the left anterior descending artery. In the EDTA group, we slowly infused 50 mL of the EDTA-based chelating solution into the intracoronary artery through the over-the-wire balloon catheter for 5 min after reperfusion (Fig. 3). Defibrillator pads were placed on the chest wall. We monitored the ECG during either ischemia or reflow. If ventricular fibrillation or ventricular tachycardia occurred, we performed electrical cardioversion with biphasic direct-current shock of 150 J.

### Area at risk, infarct size, and salvage percentage

The first 10 pigs in this study underwent 60 min of coronary occlusion, followed by 20 min of reperfusion. At 50 min after coronary occlusion, 0.25% Evans blue dye was administered into the coronary circulation via the guiding catheter to delineate the area at risk (AAR) of infarction. After 20 min of reperfusion, we administered 2% triphenyltetrazolium chloride (TTC) solution to the left anterior descending artery at the occlusion level to differentiate necrotic from viable myocardium. The pigs were euthanized under deep isoflurane anesthesia by intravenous bolus injection of KCl. The heart was exposed via a median sternotomy, immediately excised, and sliced into five to six transverse slices, and digital images were obtained. AAR and infarcted segment (IFS) in each slice were quantified from the digital photographs using image analysis software (ImageJ, NIH). Analyses of AAR and IFS were performed by a blinded person without knowledge of the study group. Subsequently, we calculated the salvage percentage using the following formula: salvage percentage (%) = (AAR-IFS) × 100/AAR.

### Immunohistochemisty

For identification of the cardiac structure, myocardial tissues from normal, AAR, and infarcted cores were obtained and sliced into 25 μm sections. The tissues were washed with TBS-Tween 20 buffer and antibody retrieval was performed. The sections were incubated with mouse anti-heavy chain cardiac myosin (ab50967, Abcam; 1:100). Then, the prepared sections were incubated with the wheat germ agglutinin (Alexa Fluor 555, Invitrogen; 1:100) conjugate and donkey anti-mouse IgG (Alexa Fluor 488, Invitrogen; 1:100) for 1 h at room temperature. Nuclei were stained with DAPI and images were observed with confocal microscopy (LSM 710).

### Determination of altered mitochondria ultrastructure by electron microscopy

We obtained tissues from the non-ischemic zone, AAR without necrosis, and infarcted core of the harvested hearts and examined altered mitochondrial ultrastructure by electron microscopy. Briefly, tissues were fixed overnight in a mixture of cold 2.5% glutaraldehyde in 0.1 M phosphate buffer (pH 7.2) and 2% paraformaldehyde in 0.1 M phosphate or cacodylate buffer (pH 7.2). The tissues were post-fixed for 1.5 h in 2% osmium tetroxide in 0.1 M phosphate or cacodylate buffer at room temperature. The samples were washed with deuterated H_2_O_2_, and dehydrated through a graded ethanol series(50%, 60%, 70%, 80%, 90%, 95%, and 100% (× 2)), infiltrated using propylene oxide and EPON epoxy resin mix (Embed 812, Nadic methyl anhydride, Poly/Bed 812, dodecenylsuccinic anhydride, and dimethylaminomethyl phenol; Electron Microscopy Polysciences, USA), and embedded with epoxy resin. The epoxy resin-mixed specimens were loaded into capsules and polymerized at 38 °C for 12 h and 60 °C for 48 h. Sections for light microscopy were cut at 500 nm and stained with 1% toluidine blue for 45 s on a hot plate at 80 °C. Thin sections were made using an ultramicrotome (RMC MT-XL) and collected on a copper grid. Appropriate areas for thin sectioning were cut at 65 nm and stained with saturated 6% uranyl acetate and 4% lead citrate before examination with a transmission electron microscope (JEM-1400; Japan) at 80 kV^20^.

In each of the samples, we counted the mitochondria in a unit area and classified the state of the mitochondria as normal (intact outer membrane and normal size), edematous (intact outer membrane but swollen), or burst (breakage of the outer membrane). Subsequently, we compared the number of mitochondria in each state between the two groups.

### Reactive oxygen species, calcium, and magnesium concentration

We obtained myocardial ischemic tissues at 0, 5, 10, and 20 min after reperfusion by punch biopsy. Tissue specimens were procured from both the ischemic area and the normal area. The specimens were immediately immersed in paraformaldehyde. Myocardial Sections (5 μm) were incubated with MitoSOX™ Red (5 mM, Invitrogen) for 15 min (room temperature and darkness) to assess ROS production by confocal microscopy (LSM800; excitation wavelength 488 nm, emission wavelength 565 nm). Nuclei were identified using DAPI (1 mg/mL).

Samples for calcium and magnesium were centrifuged at 12,000 rpm for 1 min. Five microliters of the supernatant was taken and mixed with an equal volume of 10% trichloroacetic acid (TCA) and incubated for 5 min at room temperature. Calcium content was measured using a calcium assay kit (QuantiChrom™, Bioassay Systems, DICA-500) and the quantitative analysis of magnesium ions was performed using a commercial magnesium assay kit (QuantiChrom™, Bioassay Systems, DIMG-250). All procedures were carried out according to the manufacturer’s protocol, and the protein content was determined using the Bradford Protein Assay to compensate for the concentration levels of calcium and magnesium.

### Echocardiogram

We performed echocardiography (Philips, ie-33, Sector Array Probe S12-4) at baseline, post-MI (after reperfusion), and on day 28 in 20 pigs to evaluate left ventricular remodeling (Fig. 3). We measured the left ventricular end-diastolic diameter (LVEDD) and left ventricular end-systolic diameter (LVESD) at a parasternal long axis view. We also assessed the left ventricle end-diastolic volume (LVEDV) and left ventricle end-systolic volume (LVESV) by volumetry at a subxiphoid apical long axis view. The volumetric left ventricular systolic function was evaluated by calculating the ejection fraction as follows: EF(%) = (LVEDV – LVESV) × 100/LVEDV. We measured the total left ventricle (LV) perimeter and the akinetic segment of the LV as an infarcted length on day 28. We calculated the percentage of an infarcted segment compared to the total LV perimeter.

### Statistical analysis

All continuous variables were expressed as the mean ± SD. All values in ImageJ were measured three times to ensure accuracy. We analyzed the difference in mean values between the two groups using unpaired two-sample Wilcoxon test. The effect of two groups (control and EDTA) on repeated measurements within an animal was compared with a two-way ANOVA test. Statistical analyses and graphical visualization were performed using R program version 3.3.2 and “ggpubr package” in the R program (The R Foundation for Statistical Computing, Vienna, Austria; http://www.R-project.org). A *p* value < 0.05 was considered statistically significant.

### Ethic approval

This study was approved by the Institutional Animal Care and Use Committee(IACUA) of Seoul National University Bundang Hospital and was performed in accordance with the Guide for the Care and Use of Laboratory Animals from the Institute of Laboratory Animals Resources. The study was conducted in compliance with the ARRIVE guidelines.

## Data Availability

Materials, data, and associated protocols will be made promptly available to readers on request.

## Abbreviations

AAR: Area at risk
EDTA: Ethylenediaminetetraacetic acid
IFS: Infarcted segment
IR: Ischemia–reperfusion
LVEDD: Left ventricular end-diastolic diameter
LVESD: Left ventricular end-systolic diameter
LVEDV: LV end-diastolic volume
LVESV: LV end-systolic volume
LV EF: Left ventricular ejection fraction by volumetric calculation
MI: Myocardial infarction
mPTP: Mitochondrial permeability transition pore
ROS: Reactive oxygen species
STEMI: ST segment elevation myocardial infarction
TTC: Triphenyltetrazolium chloride
