# Author Correction: Attenuation of ischemia–reperfusion injury by intracoronary chelating agent administration

**DOI:** 10.1038/s41598-023-37877-5

**Published:** 2023-07-03

**Authors:** Donghoon Han, Si-Hyuck Kang, Chang-Hwan Yoon, Tae-Jin Youn, In-Ho Chae

**Affiliations:** 1 Department of Internal Medicine, Cardiovascular Center, Hallym University Kangnam Sacred Heart Hospital, Seoul, Korea; 2 Department of Internal Medicine, Seoul National University College of Medicine, Cardiovascular Center, Seoul National University Bundang Hospital, 82 Gumiro 173, Bundang, Seongnam, Republic of Korea

Correction to: *Scientific Reports* 10.1038/s41598-022-05479-2, published online 08 February 2022

The original version of this Article contained an error in the Acknowledgements section.

“This work was supported by a grant from the Korean Health Technology R&D Project, Ministry of Health and Welfare, Republic of Korea (HI20C1456 and HI19C0655), and by a grant from the SNUBH Research Fund (14-2017-012). T.E.M was supported from Seoul National University Hospital Biomedical Research Institute.”

now reads:

“This work was supported by a grant from the Korean Health Technology R&D Project, Ministry of Health and Welfare, Republic of Korea (HI19C0655), by the Korea Medical Device Development Fund grant funded by the Korean government (Project Number: RS-2020-KD000229), and by a grant from the SNUBH Research Fund (14-2017-012). T.E.M. was supported by Seoul National University Hospital Biomedical Research Institute.”

The original Article has been corrected.

